# Effects of Environmental Non-Essential Toxic Heavy Metals on Epigenetics During Development

**DOI:** 10.3390/toxics13030167

**Published:** 2025-02-27

**Authors:** Hisaka Kurita, Kazuki Ohuchi, Masatoshi Inden

**Affiliations:** Laboratory of Medical Therapeutics and Molecular Therapeutics, Gifu Pharmaceutical University, 1-25-4 Daigaku-nishi, Gifu 501-1196, Japan; ohuchi-ka@gifu-pu.ac.jp (K.O.); inden@gifu-pu.ac.jp (M.I.)

**Keywords:** epigenetics, non-essential toxic heavy metals, Developmental Origins of Health and Disease

## Abstract

We are exposed to a variety of environmental chemicals in our daily lives. It is possible that the effects of this daily chemical exposure could accumulate in the organism in some form and influence health and disease development. The exposure effects extend throughout the human lifetime, not only after birth, but also during the embryonic period. Epigenetics is an important target for the molecular mechanisms of daily environmental chemical effects. Epigenetics is a mechanism of gene transcription regulation that does not involve changes in DNA sequence. The Developmental Origins of Health and Disease (DOHaD) theory has also been proposed, in which effects such as exposure to environmental chemicals during embryonic period are mediated by epigenetic changes, which may lead to risk for disease development and adverse health effects after maturity. This review summarizes the association between embryonic exposure and the epigenetics of well-known non-essential toxic heavy metals (methylmercury, cadmium, arsenic, and lead), a representative group of environmental chemicals. In the future, it will be important to predict the epigenetic mechanisms of unknown chemical and combined exposures. In addition, further experimental investigations using experimental animals and the accumulation of knowledge are needed to study the transgenerational effects of environmental chemicals in the future.

## 1. Introduction

We are exposed to several environmental chemicals daily. It is possible that the exposure effects may accumulate in the organism in some form and affect health and disease development. Epigenetics is a mechanism for regulating gene transcription without changes in DNA sequence. The molecules for the phenomenon of epigenetics include DNA methylation, histone modifications, and non-coding RNA [1]. Among epigenetic modifications, relatively chemically stable modifications such as DNA methylation can be accumulated [2]. The Developmental Origins of Health and Disease (DOHaD) theory has also been proposed, which states that the environment during the embryonic period influences disease development and health later in adulthood [3]. The embryonic environment would include environmental chemical exposure in addition to nutritional status [4]. Furthermore, the effects of prenatal exposure are of concern as transgenerational effects as they can affect the generation of grandchildren [5]. Epigenetic modifications, including DNA methylation, would be deeply implicated as molecular mechanisms behind these DOHaD theory effects [6]. The embryonic stage is a time of major changes in epigenetics, such as DNA methylation [7]. Therefore, changes in the prenatal environment, such as environmental chemicals during the embryonic period, could cause disturbances in epigenetic changes. A possible molecular mechanism for the effects of DOHaD is that these epigenetic modification disturbances, caused by environmental changes during the embryonic period, may persist into adulthood and be genetically imprinted as a risk factor for disease development. In view of the DOHaD theory, environmental influences, such as chemicals, continue to affect disease and health throughout life via the phenomenon of epigenetics from the embryonic period. This review summarizes the association between embryonic exposure and the epigenetics of well-known non-essential toxic heavy metals (methylmercury, cadmium, arsenic, and lead), a representative group of environmental chemicals.

## 2. Overview of Epigenetic Modifications

### 2.1. DNA Methylation

DNA methylation refers to the binding of methyl groups to nucleotides in DNA and is one of the epigenetic modifications. In mammalian DNA, the methylation of cytosine to 5-methylcytosine (5-mC) occurs primarily as a sequence of cytosine followed by guanine, which is called a CpG site. DNA methylation is generally associated with the suppression of gene expression levels. DNA methylation represses gene expression by reducing chromatin accessibility and inhibiting the function of DNA-binding proteins such as transcription factors. CpG islands are CpG-dense regions located near the 5′ transcription start site of genes and are important for regulating gene expression [8]. DNA methylation is the enzymatic formation of a covalent bond between the methyl group of the methyl group donor, S-adenosylmethionine (SAM), and the cytosine ring of the CG dinucleotide on the CpG island, forming 5-mC [9]. This reaction is catalyzed by the enzyme DNA methyltransferase (DNMT). The major isoforms of DNMT are DNMT1, DNMT3A, and DNMT3B [10]. The main role of DNMT1 is to maintain the existing methylation pattern, especially during DNA replication, and it recognizes hemimethylated DNA to maintain the methylation state. The DNMT3A and DNMT3B enzymes catalyze the new methylation of previously unmethylated regions of DNA.

On the other hand, there are two types of DNA demethylation processes: passive and active. Passive demethylation occurs by the replication-dependent dilution of 5-mC in the absence of maintenance methylation [11]. Active demethylation is performed by the TET (ten eleven translocation) enzyme, a member of the DNA hydroxylase family [12]. The TET family consists of TET1, TET2, and TET3. TET mediates DNA demethylation by oxidizing 5-mC to 5-hydroxymethylated cytosine (5-hmC). 5-hmC is oxidized to 5-formylcytosine and 5-carboxylcytosine [13]. With respect to differentiation and development, TET1 is highly expressed in embryonic stem cells (ESCs) and plays an important role in self-renewal [14]. TET1 also maintains the pluripotency of ESCs and the sustained expression of Nanog by mediating 5-mC to 5-hmC conversion or by DNMT inhibition of the DNA methylation of the promoter of the Nanog gene [14]. 5-hmC is not only a 5-mC demethylation product, but also a relatively stable epigenetic modification that regulates gene expression in a 5-mC-independent manner [15]. The target genomic regions of 5-mC and 5-hmC are probably different. 5-mC is genome-wide, except for CpG islands, enhancers, and promoters [16], while 5-hmC is highly concentrated in enhancers and gene bodies [17]. The role of 5-hmC in gene expression is not entirely clear, but it is usually present in the promoters of low-expressing genes [18]. Therefore, 5-hmC could be involved in the repression of gene expression.

### 2.2. Histone Modifications

In eukaryotes, approximately 147 bp of DNA is wrapped around an octameric complex of four core histone proteins, H2A, H2B, H3, and H4, to form the nucleosome, the basic structural unit of chromatin [19]. Histone proteins are post-translationally modified. They affect chromatin structure [20]. This causes the chromatin structure to change to either heterochromatin or euchromatin. Heterochromatin is condensed and transcriptionally inactive. Euchromatin is less condensed and transcriptionally active [21]. The chromatin states of transcriptional activity and transcriptional repression are altered by different combinations of histone post-translational modifications that occur at specific amino acid residues in the N-terminal tail. For example, the transcription start site of actively transcribed genes is usually marked by lysine acetylation (e.g., AcH3K4) and trimethylation of H3K4 (H3K4me3) at the histone H3 and H4 ends [22]. Repressed genes are often associated with H3K27me3, and heterochromatin is generally characterized by repressive H3K9me3 [23,24]. In terms of differentiation and development, histone modifications play a central role in maintaining and differentiating stem cells by altering the chromatin structure [25]. Bivalent chromatin structures consisting of both activating (H3K4me3) and repressive (H3K27me3) histone modifications are often found at promoters and enhancers that control expression of ESC developmentally regulated genes [26]. In this way, bivalent promoters can rapidly activate repressed genes during differentiation and lineage commitment [23].

Various enzymes that catalyze several histone modifications have been identified, including histone acetyltransferases (HATs) and histone deacetylases (HDACs), and histone lysine methyltransferases (HMTs) and histone lysine demethylases (HDMs). These epigenetic modifications are associated with several diseases and have a variety of effects on gene expression. The enzymes are involved, for instance, HATs, which add acetyl groups, and HDACs, which remove acetyl groups.

The 18 HDACs are divided into 4 classes [27]. Class 1 HDACs (HDAC1, 2, 3, and 8) play an important role in regulating gene expression. HDAC1 and HDAC2 are the two most studied HDACs. They are reported as the corepressors for the element-1 silencing transcription factor complex and Sin-associated protein complex, and are found in several corepressor complexes, including the nucleosome remodeling complex and histone deacetylation complex [28]. HATs are enzymes that catalyze the transfer of acetyl groups from acetyl CoA to specific lysine residues on histones and non-histone proteins, resulting in acetylation [29]. The acetylation of histone proteins alters the chromatin structure, leading to a more relaxed state that is accessible to transcription factors and regulates gene expression. The acetylation of non-histone proteins affects their stability, enzymatic activity, subcellular localization, and interactions with DNA and other proteins [30]. HATs are classified into two types, based primarily on their cellular localization. Type A HATs are primarily nuclear enzymes that acetylate nuclear histones and non-histone proteins [31]. These enzymes are classified into different families based on sequence homology and include the GNAT (general control non-repressible/GCN5-related N-acetyltransferase), MYST, and P300/CBP families [31]. B-type HATs, such as HAT1, HAT2, and HAT4, are cytoplasmic enzymes that modify free histones before they are transported to the nucleus and incorporated into newly synthesized DNA [32]. These enzymes play an important role in acetylating newly synthesized histones prior to their assembly into nucleosomes.

Among histone methylation, H3K4me1 is methylated by HMTs, including KMT2C (MLL3) and KMT7/9 (SET7/9), and removed by histone demethylases (HDM), including KDM1A (LSD1) [33]. LSD1 controls the balance between self-renewal and differentiation [34]. Modifying and removing the appropriate histone H3K4me3 is essential for stem cells to self-renew and differentiate. H3K4me3 is modified by several HMTs including MLL1 and removed by several HDMs including KDM5A and KDM5B (JARID1A/B) [35]. KMT2A, KDM5A, and KDM5B play important roles in the maintenance of the pluripotency, self-renewal, and differentiation of stem cells [36]. H3K9me2 is modified by HMT, containing G9a or G9a-like protein, and removed by HDM, containing KDM3A or KDM3B (JMJD1A/B) with Jumonji C domain [37]. G9a and GLP form a heteromeric complex and the deficiency of either reduces H3K9me2 [38]. G9a is required to maintain pluripotency [39]. KDM3A regulates self-renewal and its deficiency leads to ESC differentiation [40]. Polycomb repressive complex 2 (PRC2) is the major enzyme complex that catalyzes all three forms (me1, me2, me3) of methylated H3K27. PRC2 is mainly composed of the enhancer of zeste homolog 2 (EZH2), embryonic ectoderm development, and the suppressor of zeste 12, and EZH2 is essential for the HMT activity of PRC2 [41]. The deletion of EZH2 severely inhibits hESC self-renewal, proliferation, and differentiation [42].

### 2.3. Non-Coding RNA

In the human genome, the majority of DNA sequences (more than 97%) do not directly encode proteins. However, approximately 80% of non-coding regions are transcriptionally active in certain cell types, and the transcription of these regions generates ncRNAs, including tRNAs and rRNAs, with diverse structures and functions. miRNAs are a type of ncRNA that has been extensively studied. miRNAs are transcribed by RNA polymerase II and processed into mature miRNAs in the nucleus and cytoplasm, where they are processed into mature miRNAs [43]. Although miRNAs are functionally important and evolutionarily conserved, they play different roles in different cell types and under different conditions. Primary and precursor miRNAs are processed by RNA-binding proteins (RBPs) to produce mature miRNAs that induce Argonaute proteins, recruit RBPs, bind to target mRNAs, and repress mRNA translation [44]. miRNAs are also involved in complex regulatory mechanisms such as deadenylation, decapping, exonuclease degradation, and target cleavage [45].

## 3. Methodology

For each of the non-essential toxic heavy metal items reviewed, PubMed was used as a database. The keywords for the search were “metal name” and “epigenetic”. As a result, we found 130 reports for “mercury epigenetic”, 350 reports for “cadmium epigenetic”, 565 reports for “arsenic epigenetic”, and 495 reports for “Pb epigenetic”. We further selected papers related to perinatal exposure and development based on the abstract and the title of the paper. Articles were selected if they were written in English. As a result, 10 papers on mercury, 9 papers on cadmium, 8 papers on arsenic, and 5 papers on lead were used as references to develop the individual metal sections in Table 1 and Table 2 of this review. Other papers relevant to the introduction and discussion of the content of each item are cited separately in the text.

## 4. Effects of Non-Essential Toxic Heavy Metals During Development on Epigenetics

Non-essential toxic heavy metals (mercury, cadmium, arsenic, and lead) are focused on in this review. The effects of non-essential toxic heavy metal exposure during development on epigenetics mechanisms are summarized as follows (Table 1 and Table 2).

### 4.1. Mercury

Currently, there is concern about the effects of methyl mercury (MeHg) exposure on the fetus. This is caused by pregnant women consuming large fish species in which MeHg has bioaccumulated and accumulated. There are several experimental reports on the mechanisms by which prenatal MeHg exposure affects higher brain function. For example, the exposure of rats to MeHg 1 ppm via drinking water between gestational day 20 and postnatal day (PND) 1 induced neurodegeneration and the decrease in tropomyosin receptor kinase (Trk) A pathway and activity-regulated cytoskeleton-associated protein (Arc) expression [46]. Rats exposed to MeHg 5 ppm via drinking water from gestational day1 to PND21 showed a reduction in the PSD95 level at PND21 and PND36 [47]. Mice exposed to MeHg 0.5 mg/kg/day via drinking water from gestational day 7 until day 7 after delivery showed anxiety-like behavior and a decrease in brain-derived neurotrophic factor (BDNF) expression in 12-week-old male offspring [48]. Epigenetics is considered important for this mechanism. The epigenetic effects of MeHg include a positive correlation between prenatal MeHg exposure levels and the DNA methylation of *NR3C1* of saliva in 7-year-olds, which was reported in a Seychellois epidemiological report [49]. Meta-analyses have reported that perinatal MeHg exposure correlates with DNA methylation changes in *MED31*, *GRK1*, and *GGH* genes in child blood [50]. In experimental reports, anxiety-like behavior has been observed in mice administrated with prenatal MeHg exposure (0.5 mg/kg/day via drinking water from gestational day 7 until day 7 after delivery). In addition, this report showed epigenetic changes, such as an increase in DNA methylation and H3K27me3 as well as a decrease in AcH3, in the BDNF gene of 12-week-old male offspring [48]. An increase in DNA methylation and a decrease in histone H3 acetylation (AcH3) in the fetal cerebral cortex due to prenatal MeHg exposure have been reported [51]. Furthermore, in LUHMES cells, immortalized cells derived from the human fetal brain, exposure to MeHg during neuronal differentiation caused an increase in DNA methylation, a decrease in AcH3 and histone H3K14 acetylation (AcH3K14), and an increase in histone H3K27me3, with an increase in the respective epigenetics-modifying enzymes DNMT1, HDAC3, and HDAC6. And these epigenetic changes could be involved in MeHg-induced neurite outgrowth suppression [51]. The exposure of LUHMES cells to MeHg during neurodifferentiation decreased *TH*, *NR4A1*, *SYP*, and *DLG4* gene expression. The decreased expressions have been implicated in an increase in H3K27me3 in *TH* promoter, an increase in DNA methylation and H3K27me3 and a decrease in AcH3K9 and AcH3K14 in *NR4A1* promoter, an increase in H3K27me3 and a decrease in AcH3 in *SYP* promoter, and an increase in H3K27me3 in *DLG4* promoter [52,53,54]. These alterations of gene expression could be involved in the suppression of neurite outgrowth and neuronal spike activity by MeHg during neuronal differentiation and development [52,53,54]. There are few reports on developmental MeHg exposure and microRNA (miRNAs). An epidemiological report suggested the association between MeHg exposure levels and the reduced expression of *miR-151-5p*, *miR-10a*, *miR-193b*, *miR-1975*, *miR-423-5p*, *miR-520d-3p*, *miR-96*, *miR-526a* + *miR-518d-5p* + *miR-520c-5p*, and the *let-7* family (i.e., *let-7a*, *let-7b*, *let-7c*, *let-7d*, *let-7g*, and *let-7i*) in the placenta [55]. In addition, transgenerational effects of MeHg exposure have been reported. In a study using zebrafish, DNA methylation in the F1 and F2 generations from females exposed to MeHg at F0 was examined, and although there were no genes with variable DNA methylation in common from F0 to F2, four genes (*CR556710.1*, *FP 102191.1*, *taar20p*, and *KCNJ2*) were found [56]. In addition, a transgenerational effect of abnormalities in glucose metabolism due to prenatal MeHg exposure has been reported in mice [57].

### 4.2. Cadmium

Cadmium (Cd) occurs in many industrial processes, such as battery production and zinc and iron smelting, and is found in tobacco [58]. Cd has been implicated in nephrotoxicity, heart disease, and cancer [59]. These toxic effects have mainly been reported in adults. On the other hand, it has also been suggested that gestational Cd exposure causes fetal growth retardation [60]. Epigenetic changes due to prenatal Cd exposure have been reported. An epidemiological report has shown maternal blood Cd levels after DNA hypomethylation of the *ATP9A* gene in cord blood [61]. Cd exposure during gestation has also been associated with low body weight in girls and low DNA methylation in the differentially methylated region (DMR) of the *PEG3* gene [62]. In addition, DNA methylation of DMRs of *IGF2R*, *KvDMR*, *SNURF*/*SNRPN*, and *GNASXL* in the cord blood of Cd-exposed children was altered, mainly in the maternal imprinting control regions, and the DMRs that were the most frequently altered among these were those involved in BMI adjustment, atrial fibrillation, and hypertension [63]. Elevated maternal Cd exposure levels correlated with decreased DNA methylation at the *PCDHAC1* gene promoter, which is important for fetal growth in the placenta [64]. A recent study also investigated whether epigenetic changes associated with prenatal Cd exposure persist from birth to childhood. As a result, some differences in DNA methylation in cord blood were reported, which appeared to be associated with prenatal Cd exposure at age 9 years. Interestingly, these epigenetic changes were found to be mainly the result of Cd exposure during pregnancy, rather than long-term Cd exposure during childhood [65]. In an experimental report on DNA methylation, prenatal exposure to Cd 10 ppm via drinking water from weaning to mating, and to Cd 50 ppm via drinking water during the whole pregnancy period until day 20 of pregnancy, increased the expression of DNMT3A in rats, which is involved in de novo CpG methylation, and increased DNA methylation in *GR* in the liver of male offspring [66]. For histone modifications, in an in vitro model of human ES cell cardiomyocyte differentiation, 0.15 and 1.5 µM of Cd exposure from differential day 0 to day 2 resulted in an increase in histone H3K27me3 [67]. The exposure of Cd 0.16 mM for 24 h reduced histone H3K27me1 levels in mouse ES cells [68]. An increase in miR*-1537* levels was associated with the Cd level of the placenta in an epidemiological study [55]. The potential for transgenerational effects due to Cd has also been reported. In F1 rats exposed to Cd during fetal life, a decrease in progesterone and the associated expression of the steroidogenic enzyme *StAR* mRNA in ovarian granulosa cells, as well as an increase in *miR-27a-3p* and the *miR10b-5p* upregulation of *StAR* expression, was observed [69]. These decreases in *StAR* expression and increases in *miR-27a-3p* and *miR10b-5p* expression were also observed in F2 [69].

### 4.3. Arsenic

Arsenic (As) is a known carcinogen and there is concern about the issue of embryonic exposure and health effects in adults due to high levels of exposure via drinking water in some areas, such as Bangladesh [70]. In the methionine cycle, the amino acid L-methionine is converted into S-adenosylmethionine (SAM) by SAM synthase [71]. SAM is a universal methyl donor and is essential for DNA and histone methylation [9,71]. Arsenic metabolism could deplete SAM and limit the activity of DNMTs [72]. In DNA methylation, negative correlations have been reported between the level of prenatal As exposure and DNA methylation of *ESR1* and *PPARGC1A* in cord blood DNA [73]. It has also been reported that there is a correlation between the level of As exposure and the DNA methylation of *LINE-1* and *p16* in fetal leukemic cells [74]. And it has been reported that prenatal As exposure increases DNA methylation in the promoter region of p53 and promotes carcinogenesis [75]. There is an increased incidence of liver cancer after maturation in mice exposed to NaAsO_2_ 85 ppm via drinking water from gestational day 8 to day 18, with increased H3K27me3 in *Fabp4*, and increased H3K4me2 in *Slc25a30* in liver [76]. Suppressed TET activity and decreased hydroxy-DNA methylation were seen in a mouse fetal brain at embryonic day 18, and anxiety-like behavior was seen in adulthood after exposure to NaAsO_2_ (15 mg/L) via drinking water from gestational day 1 to day 18 [77]. On the other hand, reports on histone modifications showed an increase in H3K4me3 and histone methyltransferase (MLL) in the male and female dentate gyrus, a decrease in histone demethylase (KDM5B) in the male dentate gyrus, an increase in AcH3K9 and histone acetyltransferase (GCN5) in the male dentate gyrus, a decrease in AcH3K9K9 and an increase in HDAC1 and HDAC2 in the female dentate gyrus, an increase in H3Kme3 and MLL in the male frontal cortex, and a decrease in AcH3K9K9, GCN5, and PCAF in the male frontal cortex after exposure to Na_3_AsO_4_ 50 ppb via drinking water 10 days prior to mating, during gestation, and until pups were weaned at approximately postnatal day 23 [78]. Arsenic (As_2_O_3_ 0.93 mM) exposure for 24 h reduced levels of histone H3K27me1 in mouse ES cells [68]. Reports on miRNAs include the increased expression of 12 miRNAs (*let-7a*, *miR-107*, *miR-126*, *miR-16*, *miR-17*, *miR-195*, *miR-20a*, *miR-20b*, *miR-26b*, *miR-454*, *miR-96*, and *miR-98*) in the neonatal cord blood when maternal As exposure levels were high [79]. In addition, the transgenerational effects of arsenic exposure have been reported. An increased incidence of liver cancer in mice exposed to NaAsO_2_ 85 ppm via drinking water from gestational day 8 to day 18 has been reported to be caused not only in F1 but also in F2 [80]. Characteristics of arsenic-induced methylome changes in F1 sperm are re-established in both the paternal and maternal genomes of F2 embryos after epigenetic reprogramming [81].

### 4.4. Lead

Human exposure to lead (Pb) occurs through the inhalation of air contaminated with lead dust, the ingestion of contaminated food and water, and direct contact through the skin [82]. Pb has a high gastrointestinal absorption rate and can cross the blood–brain barrier, making the fetus and young children particularly susceptible to neurotoxicity [83]. Prenatal Pb exposure has been reported to be associated with hematopoietic toxicity and with neurodevelopmental disorders and severe cognitive impairment in adulthood. The epidemiological report has suggested that the effects of perinatal Pb exposure on global DNA methylation levels in cord blood leukemia show an inverse dose–response relationship between maternal lead levels in the patella and *LINE-1* and *Alu* DNA methylation levels [84]. Strong inverse associations with perinatal Pb exposure were observed in the levels of CpG methylation in cord blood of the *DNHD1* and *CLEC11A* genes in the human brain [85]. In an experimental report, low DNA methylation of the *Chd7* gene was observed in 20-week-old mouse brain due to exposure of 300 ppm Pb via drinking water embryonic from day 8.0 to 10.5 [86]. Furthermore, male mice exposed to 0.2% Pb on postnatal days 1–20 showed reduced levels of MeCP2, DNMT1, H3K9Ac, and H3K4me2, and increased levels of H3K27me3, in the brain across the lifespan after Pb exposure [87]. An increase in the miR-651 level and decreases in *let-7f*, *miR-146a*, *miR-10a*, and *miR-431* were associated with the Pb level of the placenta in an epidemiological study [55]. Possible transgenerational effects of Pb exposure have also been reported. Maternal blood lead levels and DNA methylation status could directly affect blood lead levels in children and grandchildren [88]. Behavioral abnormalities were observed in F3 in mice due to gestational lead exposure, but no detailed analysis, including epigenetics, has been conducted [89].

**Table 1:** Epidemiological studies of effects of developmental non-essential toxic heavy metal exposure on epigenetic modifications in children.

Metals	Study Design	Samples for Exposure Assessment	Information and Number of Subjects	Study Name and Area	Target Genes and Types of Epigenetics Modifications	References
Hg	cohort	total Hg of maternal hair DNA from the children’s saliva	saliva from 7 years-old children (n = 406)	Seychelles Child Development Study (Seychelles)	increase in DNA methylation in *GRIN2B*, *NR3C1*	Ulloa et al., 2021, *Environ. Int.* [49]
Hg	meta-analysis from several cohorts	total Hg in cord blood, maternal hair, or maternal blood DNA from cord or child blood	cord or child blood from 7 to 8 years-old children (n = 794)	Avon Longitudinal Study of Parents and Children (United Kingdom) Hokkaido Study on Environment and Children’s Health, Sapporo cohort (Japan) Environment and Childhood Project (Spain) KOREAN Exposome (Korea) Project VIVA (United States) Mother, Father and Child Cohort Study (Norway) Mother-Child Cohort in Crete (Greece)	increase in DNA methylation in *MED31*, *GGH*, *GRK1*	Lozano et al., 2022, *Environ. Res.* [50]
Hg	cohort	Hg and miRNA from Placenta	placenta (n = 110)	National Children’s Study (United States)	decrease in miRNA in miR-151-*5p*, *miR-10a*, miR-193b, miR-1975, *miR-423-5p*, *miR-520d-3p*, *miR-96*, *miR-526a* + *miR-518d-5p* + *miR-520c-5p*, *let-7* family (i.e., *let-7a*, *let-7b*, *let-7c*, *let-7d*, *let-7g*, and *let-7i*)	Li et al., 2015, *Epigenetics* [55]
Cd	cohort	Cd in maternal blood DNA in cord blood	cord blood (n = 364)	Mothers and Children’s Environmental Health multicenter prospective cohort study (Korea)	decrease in DNA methylation in *ATP9A*	Park et al., 2022, *Environ Res* [61]
Cd	cohort	Cd in maternal blood DNA in cord blood	cord blood (n = 319)	Newborn Epigenetic Study (United States)	increase in DNA methylation in *MEG3* in male increase of DNA methylation in *PEG3* in female	Vidal et al., 2015, *BMC Pharmacol Toxicol* [62]
Cd	cohort	Cd in maternal blood DNA in cord blood	cord blood (n = 19)	Newborn Epigenetic STudy (United States)	increase in DNA methylation in *IGF2R*, *KvDMR*, *SNURF*/*SNRPN*, *GNASXL* decrease in DNA methylation in *GNASXL*	Cowley et al., 2018, *Environ Health Perspect* [63]
Cd	cohort	toenail from mothers and newborns placenta	placenta (n = 94)	Rhode Island Child Health Study (United States)	decrease in DNA methylation in *PCDHAC1*	Everson et al., 2016, *Reprod Toxicol* [64]
Cd	cohort	Cd in maternal blood DNA in cord blood	cord blood from 9 years-old children (n = 81)	mother–child cohort in Matlab (Bangladesh)	increase in DNA methylation in *GSTT1*, *SAMD11* decrease in DNA methylation in *AURKC*, *LY6G5C*, *TACSTD2*, *HLA-DQB2*, *NCRNA00200*	Gliga et al., 2022, *Environ Int* [65]
Cd	cohort	Cd and miRNA from Placenta	placenta (n = 110)	National Children’s Study (United States)	increase in miRNA in miR-1537	Li et al., 2015, *Epigenetics* [55]
As	cohort	arsenic in maternal urine DNA in cord blood	infant (n = 134)	New Hampshire Birth Cohort Study (United States)	decrease in DNA methylation in *ESR1*, *PPARGC1A*	Koestler et al., 2013, *Environ Health Perspect* [73]
As	cohort	arsenic in drinking-water and urine DNA in cord blood	cord blood (n = 113)	prospective birth cohort recruited in Sirajdikhan Upazila (Bangladesh)	increase in DNA methylation in *LINE-1*, *p16*	Kile et al., 2012, *Environ Health Perspect* [74]
As	cohort	arsenic in drinking-water DNA in cord blood	cord blood (n = 55)	Thailand	increase in DNA methylation in *p53*	Intarasunanont et al., 2012, *Environ Health* [75]
As	cohort	arsenic in maternal urine or drinking water DNA in cord blood	cord blood (n = 40)	Biomarkers of Exposure to Arsenic prospective pregnancy cohort (Mexico)	increase in let-7a, miR-107, miR-126, *miR-16*, miR-17, *miR-195*, *miR-20a*, *miR-20b*, *miR-26b*, *miR-454*, *miR-96*, and *miR-98*	Rager et al., 2014, *Environ Mol* Mutagen [79]
Pb	cohort	Pb in maternal bone DNA in cord blood	cord blood (n = 103)	Early Life Exposures in Mexico to Environmental Toxicants study (Mexico)	decrease in DNA methylation in *LINE-1*, *Alu*	Pilsner et al., 2009, *Environ Health Perspect* [84]
Pb	cohort	Pb in maternal blood DNA in cord blood	cord blood (n = 268)	Project Viva (United States)	decrease in DNA methylation in *CLEC11A* decrease of DNA methylation in DNHD1 in female	Wu et al., 2017, *Environ Health* Perspect [85]
Pb	cohort	Pb and miRNA from Placenta	placenta (n = 110)	National Children’s Study (United States)	decrease in miRNA in *let-7f*, *miR-146a*, *miR-10a*, and *miR-431* increase if miRNA in *miR-651*	Li et al., 2015, *Epigenetics* [55]

**Table 2:** Experimental studies of effects of developmental non-essential toxic heavy metal exposure on epigenetic modifications.

Metals	Study Design	Exposure Condition	Information of Samples	Target Genes and Types of Epigenetics Modifications	References
Hg	C57BL/6 mouse	pregnant mice exposed to MeHg (CH3HgOH) 0.5 mg/kg/day via drinking water from gestational day 7 until day 7 after delivery	male 12-week-old offspring	increase in DNA methylation in *BDNF* decrease in AcH3 in *BDNF* increase in H3K27me3 in *BDNF*	Onishchenko et al., 2008, *J. Neurochem.* [48]
Hg	C57BL/6 mouse	pregnant mice exposed to MeHg (CH_3_HgCl) 3 mg/kg/day via single oral administration from gestational day 12 to day 14.	fetal cortex at embryonic day 19	increase in DNA methylation increase in DNA, AcH3K14 increase in DNMT1 increase in DNMT1, HDAC6	Go et al., 2021, *Arch Toxicol* [51]
Hg	LUHMES cells	MeHg (CH3HgCl) 1 nM from differentiational day 2 to day 8	cell culture	increase in DNA methylation decrease in AcH3, AcH3K14 increase in H3K27me3 increase in H3K27me3, DNMT3A, DNMT3B decrease in HDAC3, HDAC6	Go et al., 2021, *Arch Toxicol* [51]
Hg	LUHMES cells	MeHg (CH_3_HgCl) 1 nM from differentiational day 2 to day 8	cell culture	increase in H3K27me3 in *TH*	Go et al., 2018, *Biochem Biophys Res Commun* [52]
Hg	LUHMES cells	MeHg (CH3HgCl) 1 nM from differentiational day 2 to day 8	cell culture	increase in DNA methylation in *NR4A1* decrease in AcH3K9K9, AcH3K14 in *NR4A1* increase in H3K27me3 in *NR4A1*	Go et al., 2023, *Toxicol Lett* [53]
Hg	LUHMES cells	MeHg (CH_3_HgCl) 1 nM from differentiational day 2 to day 8	cell culture	increase and decrease in DNA methylation in *SYP*, *DLG4* decrease in AcH3 in *SYP* increase in H3K27me3 in *SYP*, *DLG4*	Kurita et al., 2024, *J Appl Toxicol* [54]
Cd	Wistar rat	Cd (CdCl_2_) 10 ppm via drinking water from weaning to mating, and Cd 50 ppm via drinking water whole pregnancy period until day 20 of pregnancy	liver of pups at gestational day 20	increase in DNA methylation *in GR* in male increase in DNMT3a in male	Castillo et al., 2012, *PLoS One* [66]
Cd	human cardiomyocyte differentiated from embryonic stem cells	Cd (CdCl_2_) 0.15–1.5 uM from differentiational day 0 to day 2	cell culture	increase in H3K27me3	Wu et al., 2022, *Environ Health Perspect* [67]
Cd	mouse embryonic stem cells	Cd (CdCl_2_) 0.16 mM for 24 h	cell culture	decrease in H3K27me1	Gadhia et al., 2012, *Toxicol Lett* [68]
As	C3H/HeN mouse	pregnant mice exposed to As (NaAsO_2_) 85 ppm via drinking water from gestational day 8 to day 18.	liver of 74-week-old offspring	increase in H3K9me2 in *Fabp4* increase in H3K4me3 in *Slc25a30*	Nohara et al., 2012, *Toxicol Sci* [76]
As	CD-1 mouse	pregnant mice exposed to As (NaAsO_2_) 15 mg/L via drinking water from gestational day 1 to day 18.	fetal brain at embryonic day 18	decrease of 5-hmC decrease in TET activity	Lv et al., 2021, *Ecotoxicol Environ Saf* [77]
As	C57BL/6 mouse	female mice exposed to As (Na_3_AsO_4_) 50 ppb via drinking water 10 days prior to mating, during gestation, and until pups were weaned at approximately postnatal day 23	frontal cortex and dentate gyrus from 70-day-old offsprings	increase in H3K4me3 and histone methyltransferase (MLL) in male and female dentate gyrus decrease in histone demethylase (KDM5B) in male dentate gyrus increase in H3Kme3 and MLL in male frontal cortex increase in AcH3K9 and histone acetyltransferase (GCN5) in male dentate gyrus decrease in AcH3K9K9 and decrease in AcH3K9, HDAC2 in female dentate gyrus decrease of AcH3K9 and GCN5, PCAF in male frontal cortex	Tyler et al., 2015, *Toxicol Appl Pharmacol* [78]
As	mouse embryonic stem cells	As (As2O_3_) 0.93 mM for 24 h	cell culture	decrease in H3K27me1	Gadhia et al., 2012, *Toxicol Lett* [68]
Pb	C57BL/6 mouse	Pb (Pb-acetate) 300 ppm via drinking water embryonic day 8.0 to 10.5	frontal cortex from 20-weeks-old offsprings	decrease in DNA methylation in *Chd7*	Hill et al., 2015, *Behav Neurol* [86]
Pb	C57BL/6 mouse	male mice exposed to 0.2% Pb (Pb-acetate) from postnatal day 1 to 20 through the drinking water of the dam.	brains at postnatal day 20, 180, 270, 540, and 700	decrease in MeCP2, DNMT1,H3K9Ac and H3K4me2 increase in H3K27me3	Eid et al., 2016, *Alzheimers Dement* (Amst) [87]

### 4.5. Possible Mechanisms of Non-Essential Toxic Heavy Metals on Epigenetic Modifications

**Figure 1:**
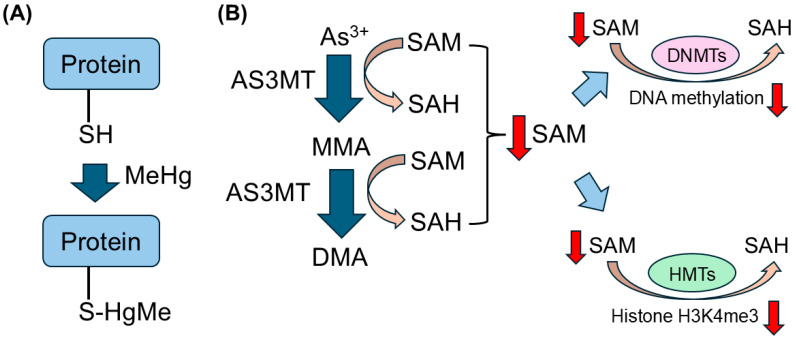
Possible mechanisms of non-essential toxic heavy metals in epigenetic modifications. (**A**) MeHg and Cd are electrophilic molecules and have been reported to form direct adducts with cysteine residues on proteins. Adduct formation on cysteine residues can inhibit the enzymatic activity of the protein itself and cause conformational changes. (**B**) As3+ is methylated by arsenic (+3 oxidation state) methyltransferase (AS3MT) and converted into methylarsonic acid (MMA) and dimethylarsinic acid (DMA), which are then excreted. In this process, SAM is consumed as a methylation donor for methylation by AS3MT. Since SAM also acts as a methylation donor during methylation by DNMTs and HMTs, the depletion of SAM associated with As metabolism could suppress methylation reactions by DNMTs and HMTs and DNA methylation could affect histone methylation. Red arrows mean the decrease of individual molecules.

In this review, we present reports on epigenetic changes in non-essential toxic heavy metals such as Hg, Cd, As, and Pb, and we will discuss the mechanisms involved (Figure 1). MeHg and Cd are electrophilic molecules and have been reported to form direct adducts with cysteine residues on proteins [90,91]. Adduct formation on cysteine residues can inhibit the enzymatic activity of the protein itself and cause conformational changes. It has been reported that the electrophilic NO forms adducts with the epigenetic modification enzymes DNMT3B and HDAC, inhibiting their enzymatic activities and altering their epigenetics [92]. It has been reported that the electrophilic nitric oxide (NO) forms adducts with the epigenetic modification enzymes DNMT3B and HDAC6, inhibiting their enzymatic activities and altering their epigenetics [93,94]. Although there have been no reports of MeHg or Cd directly inhibiting enzymes involved in epigenetic modifications by forming adducts with them, this is one of possible mechanisms. In addition, when As is metabolized, trivalent As is methylated by arsenic (+3 oxidation state) methyltransferase (AS3MT) and converted into methylarsonic acid (MMA) and dimethylarsinic acid (DMA), which are then excreted. In this process, SAM is consumed as a methylation donor for methylation by AS3MT [72]. Since SAM also acts as a methylation donor during methylation by DNMTs and HMTs, the depletion of SAM associated with As metabolism could suppress methylation reactions by DNMTs and HMTs and DNA methylation could affect histone methylation [72]. On the other hand, there is little mechanistic knowledge of the direct effect of Pb on epigenetic enzymes and epigenetic modifications.

## 5. Discussion

**Figure 2:**
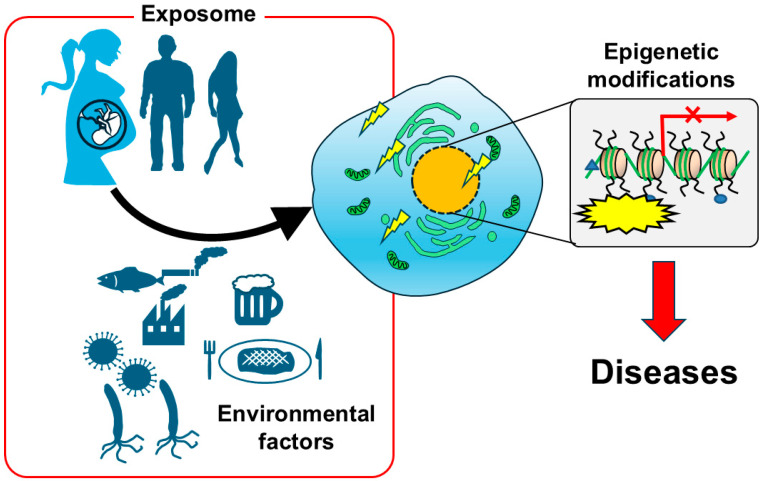
Epigenetics should be related to exposome-induced diseases. Lifelong environmental exposure effects of “exposome” would be thought to accumulate in cells as epigenetic disturbances, leading to disease onset.

There is concern about the relationship between prenatal exposure to environmental chemicals such as non-essential toxic heavy metals in the environment and health effects and diseases in adulthood [3]. One important molecular mechanism is epigenetics, although the mechanism is not clear. Although MeHg, Cd, As, and Pb were covered in this review, the actual exposure effects on humans are considered to be combined exposure due to various environmental factors. The concept of the exposome, as the totality of exposure effects over a lifetime, has been proposed [95]. Due to the limitations in assessing the effects of combined exposure to all environmental chemicals, including non-essential toxic heavy metals, initiatives such as the prediction of molecular toxicity mechanisms are needed [96]. Epigenetics is considered to be an important mechanism behind such exposome-induced effects [97] (Figure 2). In recent years, attempts have been made at the global level to develop and construct an Adverse Outcome Pathway (AOP), which can be used as a simple and rapid toxicity prediction method based on the mechanism of action. The AOP is a pathway that represents the relationship between the toxic mechanisms at each level of toxicity, from the reaction at the molecular level that causes toxicity to the effects at the cellular, organ, and organism levels, based on current knowledge [98]. The Organisation for Economic Co-operation and Development (OECD) has initiated a program to promote the development of AOPs, and expert evaluations and OECD fairness are underway, particularly in the United States and the European Union [99]. The components of an AOP consist of a Molecular Initiating Event (MIE), which is the interaction of a chemical with its initial target molecule, a Key Event (KE), at each level from which it is derived, and an Adverse Outcome (AO), which is the final adverse effect. The AOP does not represent the effects of a specific chemical, but applies to all substances that cause a given MIE and lead to a biological adverse event AO. Understanding the relationship between KEs, KERs, and experimental data (inhibitors, gene knockout, knockdown experiments to prove causality) will lead to more accurate AOP construction. If we can construct an AOP with high accuracy, we can predict AOs from specific KEs based on the AOP. Although efforts to construct AOPs are still in the developmental stage, including in terms of their reliability and data accumulation, they can significantly reduce costs in the management and regulation of chemical substances. Furthermore, combining structure–activity relationship data for chemicals and the mode of action (MoA) with AOPs could help to predict compounds that cause toxicity, starting at the stage of new chemical synthesis. From the perspective of the exposome, which focuses on the total amount of environmental exposure over a lifetime, AOPs may also be useful as a tool for predicting and evaluating the total exposure effects of countless external and internal factors over a lifetime, including the impact of the exposome on epigenetics. Epigenetic modification enzymes such as DNMTs, HDACs, and HATs are considered to be the main targets for the effects of environmental chemicals such as heavy metals on epigenetics. We believe that clarifying the relationship between the activities of these enzymes and heavy metals may help to predict epigenetic mechanisms at work to combined exposure and other factors. Therefore, accumulated knowledge, such as that presented in this review, will be important for predicting the epigenetic mechanisms of unknown chemical and combined exposures.

**Figure 3:**
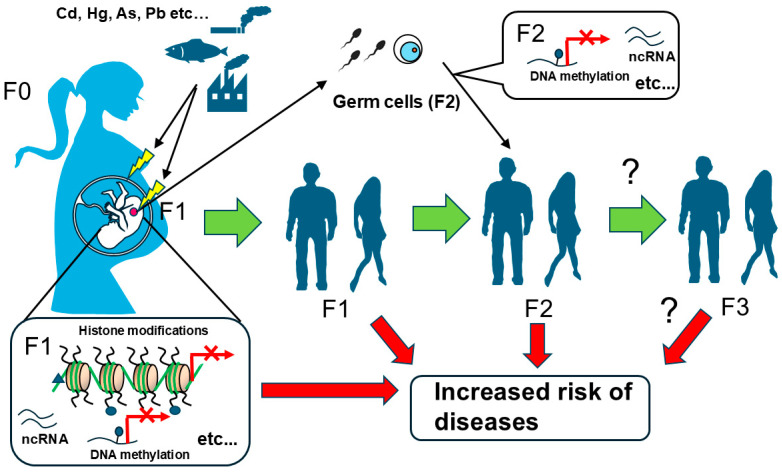
Possibility of transgenerational effects via epigenetic modification of germ cell lineage. Exposure of the mother (F0) not only directly affects the offspring (F1), but also direct exposure effects on F2 are expected, as the primordial germ cells of the future F2 generation have also started to form during the embryonic period of the F1 generation and the mechanisms or possibility of transgenerational effects in F3 would not be still clear.

Furthermore, an important issue in chemical exposure effects via epigenetics during the embryonic period involves transgenerational effects. Although the transgenerational effects of heavy metal exposure were presented in this review, there is still little knowledge available. The exposure to the mother (F0) not only directly affects the offspring (F1), but direct exposure effects on F2 are also expected, as the primordial germ cells of the future F2 generation also start to form during the embryonic period of the F1 generation [81], and mechanisms or possibility of transgenerational effects in F3 are still not still (Figure 3). It has been suggested that epigenetic modifications such as DNA methylation are important for their intergenerational effects, but the mechanism behind this is not clear. Recently, it has been experimentally demonstrated that newly acquired DNA methylation and associated phenotypes are robustly imprinted and inherited in the genome across generations [100]. Although the maintenance of this robust DNA methylation may depend on the type and extent of the stimuli that induce DNA methylation, as well as the genomic region, we believe that the report strongly supports the previously observed transgenerational effects of environmental chemical exposure via epigenetics. Further experimental investigations using experimental animals and the accumulation of knowledge are needed to study the transgenerational effects of environmental chemicals in the future.

## 6. Conclusions

This review suggests that prenatal exposure to toxic non-essential heavy metals may disrupt the epigenetics of the child and put it at risk for disease development in adulthood. It is necessary to continue to accumulate new knowledge, including about transgenerational effects, mainly through epidemiological and animal studies. In addition, research on the prediction of toxicity pathways for new chemical substances and combined exposures will be essential in the future. Regarding the prediction of toxicity pathways, epigenetic mechanisms are one of the major toxicity mechanisms, and the accumulation of knowledge on existing chemical substances, including toxic heavy metals as indicated in the contents of this review, will be useful for the construction of future toxicity prediction methods.

## Data Availability

No new data were created or analyzed in this study. Data sharing is not applicable to this article.
